# Determining Nitrate and Nitrite Content in Beverages, Fruits, Vegetables, and Stews Marketed in Arak, Iran

**DOI:** 10.1155/2014/439702

**Published:** 2014-12-25

**Authors:** Mohammad Rezaei, Ali Fani, A. Latif Moini, Parisa Mirzajani, Ali Akbar Malekirad, Mohammad Rafiei

**Affiliations:** ^1^Department of Food Safety and Hygiene, School of Public Health, Tehran University of Medical Sciences, Tehran, Iran; ^2^Arak University of Medical Sciences, Arak, Iran; ^3^Payame Noor University, Tehran, Iran

## Abstract

*Background and Objectives*. Presence of excessive nitrite and nitrate in foodstuff can have toxic and carcinogenic effects on humans. This study is aimed at measuring nitrate and nitrite in different foodstuffs available in Arak city market, Iran, in 2013. *Methods*. Totally 323 samples including stew (102 samples), beverage (116 samples), fruit (55 samples), and vegetables (50 samples) were randomly collected and analyzed according to official AOAC method 973 and ISO 6635 through spectrophotometric method. *Results*. Average concentration of nitrate and nitrite in the samples was 6.58–136.76, 1.52–38.22 mg kg^−1^ or liter, respectively. Presence of nitrate and nitrite was confirmed in all samples. High levels of nitrate and nitrite were observed in celery and ghormeh stew; and lower level of nitrate and nitrite was found in traditionally produced vinegar, verjuice, and tomato. *Conclusions*. It was found that the mean values for nitrite in investigated products were higher than ADI levels of WHO.

## 1. Introduction

Nitrate and nitrite ions are ubiquitous in the environment and naturally found in plant foods as a part of the nitrogen cycle. Vegetables are the original source of dietary nitrate; however, wide variations in nitrate levels have been observed depending on the type of vegetable, its source, conditions of cultivation, and storage [1]. The amount of available nitrate in soil (depending on the content of artificial fertilizer) appears to be major factor determining the nitrate content in vegetables [2]. An estimated daily dose of nitrates consumed by man reaches 75–100 mg, of which 80–90% come from vegetables and 5–10% come from water [3]. Nitrate in drinking water usually comes from contamination of ground water caused by fertilizer, animal, or human waste [4]. Nitrate is converted in mammalian systems (through bacterial and mammalian enzyme action) to nitrite and then reacts with amines, amides, and amino acids to form N-nitroso compound. While nitrate does not have direct carcinogenic effects on humans, nitrite and N-nitroso compounds are known to be biologically active in mammalian system [1]. Nitrate by the enzyme “nitrate reductase” (in the saliva, in the stomach, and everywhere in the human body where pH is low) converts to nitrite; nitrite reacts with hemoglobin and produces methemoglobin which enables transporting of oxygen at the cellular level. Newborn organisms are very sensitive to methemoglobinemia because they have an immature “methemoglobin reductase system” [5]. Nitrate and nitrite as the sodium or potassium salts are used as additives in meat products to provide color, taste and protect against microorganisms; however, excessive use of these substances can cause toxicity and carcinogenic effect [6]. High dietary intakes of nitrate and nitrite have been implicated in the etiology of Human gastric cancer based on epidemiology and clinical studies [7]. In the stomach, nitrite reacts with amines and amides; therefor stomach is exposed to the risk of endogenous N-nitroso compound synthesis, as stomach acid reacts with catalyses nitrosation. High nitrate intake has been found to be associated with gastric cancer in England, Colombia, Chile, Japan, Denmark, Hungary, and Italy [8]. The WHO's ADI for nitrate is of 222 mg/day for a 60 kg adult [9]. Furthermore, the toxic effects of nitrite have been reviewed in many studies [10]. This study is, therefore, aimed at measuring the nitrates and nitrites contents in some brands of stew, beverage, vegetable, and fruit which are available in market in Arak, Iran.

## 2. Materials and Methods

### 2.1. Sampling

The city under study was divided into five districts and samples from each district were collected independently and equally from different markets. Totally, 323 samples were collected including stew (*n* = 102), beverage (*n* = 116), fruit (*n* = 55), and vegetables (*n* = 50).

### 2.2. Chemical and Reagents

All of the chemical materials and standards were obtained from Merck (Darmstadt, Germany). Standards with purity of 99% were adopted for the analysis.

### 2.3. Sample Preparation

Nitrate and nitrite in fruits, vegetables, and derived products were determined based on standards of the association of analytical communities (AOAC) 973 and International Organization for Standardization (ISO) 6635 [11, 12]. The absorbances were measured after 15 minutes at 538 nm. The calibration curve was constructed by plotting the absorbance versus the concentration. Nitrate and nitrite levels in samples were reported as mg NO_2_^−^ and NO_3_^−^/kg. Distilled water as a negative control and sodium nitrite and nitrate were used as positive control twice.

### 2.4. Method Validation

#### 2.4.1. Calibration Curve

Nitrite was determined based on comparison with a standard curve, which was prepared as follows: 10, 20, 30, and 40 mL nitrite working standard solutions were added to 50 mL volumetric flasks; 2.5 mL sulfanilamide reagent was added, mixed, and preceded as above. The calibration curve was constructed by plotting the absorbance versus the concentration. It was a straight line to 1 *μ*g/mL NaNO_2_ in the final solution. Nitrite levels in samples were reported as mg NO_2_^−^/kg or liter. The standard deviation of the *y*-intercept of the calibration curve was used to calculate the limits of detection and quantification (LOD and LOQ) as follows: ICH, 1995 [13]: LOD = 3.3 × SD*y*/*S* and LOQ = 10 × SD*y*/*S*, where SD*y* is standard deviation of the *y*-intercept and *S* is slope of the regression line. For quality assurance, the 10 mg kg^−1^ nitrite standard was used as a control sample.

### 2.5. Recoveries

The accuracy of the procedure was assessed by performing recovery experiments. Vegetable, beverage, fruit, and stew samples were spiked by adding 0.5, 1, 2.5, 5, 7.5, 10, and 50 mg kg^−1^ nitrate and nitrite. The results obtained after the extraction procedure represented 100% recovery. The experimental recovery was obtained from difference between sample and spiked sample, according to the following equation: (Total analyte found − analyte originally present) × 100/analyte spike = percentage recovery (%). Calibration curve data, LOD, LOQ, and recovery values are listed in Table 4. The limits of quantification (LOQs) for beverage and stew samples were 1.4 mg kg^−1^ and 2.8 mg kg^−1^ for the fruit and vegetable. Linear spiked calibration curves for all the samples were obtained with correlation factors between 0.9883 and 0.9993. The recovery of nitrate and nitrite at these seven levels was in the range of 89–101% and of 92–109% with *n* = 3 at each spiking level, respectively.

### 2.6. Statistical Analysis

Data were normalized using the Minitab software (box-cox transformation). Statistical analysis with SAS software (version 9; SAS institute Inc., Cary, NC, USA) was carried out in order to determine significance difference (*P* values < 0.05) between product's nitrate and nitrite content.

## 3. Results and Discussion 

In this study, 323 samples of foodstuff collected from different regions in Arak city were analyzed for nitrate and nitrite levels by spectrophotometric method.

Four groups and 14 types of foodstuffs which are commonly used were sampled and the amount of nitrate and nitrite was determined (Tables 1 [Table tab2]–3). Totally, 92.8% samples had nitrite and 98.7% samples had nitrate concentrations higher than LOD level. Accordingly, the highest amounts of nitrate value in stews were found in celery, ghormeh, fesenjan, eggplant, and gheimeh stews in descending order. The highest amounts of nitrite concentration in stews were found in celery, ghormeh, gheimeh, fesenjan, and eggplant stews in descending order (Table 1). The highest amounts of nitrate concentration in beverage products were found in packed fruit juices, soft drink, traditional verjuice, and traditional vinegar in a descending order. The highest amounts of nitrite concentration in beverage products were found in the soft drink, packed fruit juices, traditional verjuice, and traditional vinegar in descending order (Table 2). The highest amounts of nitrate and nitrite concentration in fruit were found in the cantaloupe, melon, and watermelon in descending order (Table 3). The highest amounts of nitrate and nitrite concentration in vegetable were found in the cucumber and tomatoes in descending order (Table 3). In general, the highest amounts were found in the celery and ghormeh stew, respectively, and the lowest amounts were found, respectively, in traditional vinegar, traditional verjuice, and tomato in descending order. There was a significant difference between the mean concentration of nitrate and nitrite in the cantaloupe and soft drink on one hand and the other samples (Tables 1 [Table tab2]–3).

The results of this study showed that total dietary nitrate was less than the maximum level for nitrates recommended by the World Health Organization (WHO) and nitrite of some food material in Arak was more than WHO standard level. Ayaz et al. reported that the highest content of nitrate in different vegetables was found in parsley (1513.36 mg kg^−1^). On the other hand, tomato (11.06 mg kg^−1^) had the lowest levels of nitrate and the amount of nitrate in vegetables was found to be lower than the level established by the European Commission (EC) [14]. In another work, the average values of nitrates in the vegetables were less than the maximum level for nitrates recommended by the WHO and Food and Agriculture Organization (FAO); therefore the food products were safe for consumption [5]. The results are consistent with the present study. The average of nitrate and nitrite concentration in vegetable samples (spinach and leek) was less than the maximum level specified by European Commission Regulation (3000 and 0.0 mg kg^−1^, resp.) [2, 15]. A research was done in Kermanshah, Iran, by Pirsaheb et al. showing that the nitrate concentration of potato was higher than the recommended level of WHO (347.7 ± 45.4 mg kg^−1^); additionally, leafy vegetables contained the highest (673.8 ± 50/6) and nonleafy vegetables contained the lowest level of nitrate (12.5 ± 0.9 mg kg^−1^) [16]. Tamme et al. reported that the highest mean values of nitrates were found in dill, spinach, lettuce, and beetroot, and all met the health standards and codes [17]. Okafor et al. reported that some of the fruit drink samples could be unsafe for children and infants because the concentrations of nitrite in these samples were higher than the WHO's (1978) recommended acceptable daily intake (ADI) of nitrite and nitrosamines were also detected in some of the fruit juices [18]. The results of the present study are consistent with Nabrzyski and Gajewska who stated that high levels of nitrate were present in lettuce, frozen spinach, fennel, radishes, and parsley. The highest level, over the 3500 mg kg^−1^, was found in lettuce. Also, Nabrzyski and Gajewska reported that the levels of nitrate in frozen vegetables, fruits, jams, and stewed fruits were only little less than in fresh products [19]. In study by Rostkowski et al., high values of nitrate were found in the vegetables: lettuce, young red beets, radishes, dills, parsley, carrots, cucumbers, and potatoes [20]. The ADI for nitrate and nitrite is 0–3.7 mg kg^−1^ bw/day and 0–0.07 mg kg^−1^ bw/day, respectively [18]. In this study, mean levels for nitrate and nitrite in some of stew products ranged from 6.82 mg kg^−1^ to 218.3 mg kg^−1^ (Table 1). These are below the ADI for nitrate [9]. While the result of nitrite showed that 70% of gheimeh stew, 63% of eggplant stew, 72% of ghormeh stew, 100% of celery stew, and 90% of fesenjan stew samples were higher than the WHO's (1978) recommended ADI of nitrite (0.07 mg kg^−1^ body weight). There was no international data available for comparison. The mean nitrate and nitrite levels in the soft drink ranged from 12.4 to 64.2 and from 2 to 14.5 mg L^−1^, respectively (Table 2). The concentration of nitrate in the samples falls below the WHO's ADI [18]. For the children under 10 kg body weight, the levels of nitrite from the soft traditional vinegar, traditional verjuice, fruit juices, and drink water are higher than the WHO's (1978) recommended ADI (0.2 mg kg^−1^ body weight). The results regarding nitrite also showed that 46% of traditional vinegar, 16% of traditional verjuice, 61% of fruit juice, and 92% of soft drink samples had higher nitrite than the WHO's (1978) recommended ADI of nitrite. Thus, some of these beverages could be unsafe for children and infants. Children might be especially susceptible to the toxic effects of these compounds as they have low body weight, immature enzymatic system (especially for xenobiotic metabolism), and high gastric acidity (a good condition for N-nitrosamine formation) [21]. In this regard, infants' illness and death from nitrite induced methemoglobinemia have been reported by [22]. In another work, the concentration of nitrate in the fruit juices fell below the WHO's ADI (5 mg kg^−1^ body weight). Nitrate levels in the juices ranged from 2.29 ± 0.52 to 16.50 ± 2.21 and for the children under 10 kg body weight; the levels of nitrite from those fruit juices are higher than the WHO's (1978) recommended ADI of nitrite (0.2 mg kg^−1^ body weight). The results for nitrate analyses of all fruit samples are listed in Table 3, which are below the WHO's ADI level for nitrate [18]. Nitrite contents of fruits samples in Table 3 show that 75% of watermelon, 100% of cantaloupe, and 79% of melon samples have nitrite content higher than the WHO's (1978) recommended ADI of nitrite (0.07 mg kg^−1^ body weight). Thus, these fruits could be dangerous for consumption. Tamme et al. recommended that the average nitrate content in watermelon was 95 mg kg^−1^ [17]. The results of our investigations showed that (Table 3) the content of nitrates in samples of vegetables was less than the maximum level for nitrates as recommended by the WHO and FAO, 2500 mg kg^−1^ [5]. This means that the vegetables are safe for consumption. Ayaz et al. and Abo Bakr et al. reported that the tomato had a low level of nitrate: 11.06 mg kg^−1^ and 130 mg kg^−1^, respectively [14, 23]. Furthermore, Chung et al. showed that the mean concentrations of nitrate in tomato and cucumber were 57 and 110 mg kg^−1^ respectively [10]. The concentration of nitrite in the tomato samples fell below the WHO's ADI (0.07 mg kg^−1^ body weight), and 86% of cucumber had higher nitrite than the WHO's (1978) recommended ADI of nitrite (0.07 mg kg^−1^ body weight).Thus this type of vegetables could be unsafe for consumption.

## 4. Conclusion

Considering that amount of nitrate and nitrite in some of foodstuffs under study were above EU limit [2, 14], it is highly recommended that controlling measures should be taken promptly to reduce contamination with using food control systems (i.e., good agricultural practice (GAP) and hazard analysis and critical control points (HACCP)) and educating farmers. Additionally, it is suggested that studies must be conducted on nitrate and nitrite contaminations in different foodstuffs and which factors could be involved in nitrate and nitrite contamination.

## Figures and Tables

**Table 1:** Mean levels and range (mg kg^−1^) for nitrate and nitrite in some stews.

Kind of stew	Samples	Nitrate		Nitrite	
		Mean	Min–Max	Mean	Min–Max
Gheimeh stew	23	20.97^a^	6.82–56	19.99^a^	1.43–58
Eggplant stew	19	39.2^a^	2.3–116	18.17^a^	<LOD–50.4
Ghormeh stew	25	105.45^b^	15–186.6	23.8^a^	2.1–86.5
Celery stew	15	136.76^c^	56–218	38.23^a^	10.6–87.5
Fesenjan stew	20	42.26^a^	10.54–106.5	18.43^b^	3.1–36

^a,b,c^Least squares means within the same row without a common superscript differ (*P* < 0.05).

**Table 2 tab2:** Mean levels and range (mg kg^−1^) for nitrate and nitrite in some beverages.

Kind of beverages	Samples	Nitrate		Nitrite	
		Mean	Min–Max	Mean	Min–Max
Traditional vinegar	26	6.58^a^	0.64–24.2	1.51^a^	<LOD–5.5
Traditional verjuice	36	18.16^b^	<LOD–39.45	1.92^a^	<LOD–6.9
Packed fruit juices	42	37.15^a^	<LOD–137	6.8^a^	<LOD–65
Soft drink	12	34.45^c^	12.4–64.2	7.72^b^	2–14.5

^a,b,c^Least squares means within the same row without a common superscript differ (*P* < 0.05).

**Table 3:** Mean levels and range (mg kg^−1^) for nitrate and nitrite in some fruits and vegetables.

Kind of fruit	Samples	Nitrate		Nitrite	
		Mean	Min–Max	Mean	Min–Max
Watermelon	20	26.61^a^	13.95–37.7	5.5^a^	3.1–8.5
Cantaloupe	14	58.98^b^	23–103	28.36^b^	4.6–57.5
Melon	21	33.64^a^	16.28–72.5	7.65^a^	3.7–16.7
Tomatoes	21	7.82^a^	3.19–12.98	1.81^a^	<LOD–2.93
Cucumber	29	42.7^a^	12.32–88.6	9.03^a^	2.8–18.5

^a,b^Least squares means within the same row without a common superscript differ (*P* < 0.05).

**Table 4:** Characteristics of the analytical method for nitrate and nitrite.

	Calibration curve		*R* ^2^	Linearity (mg kg^−1^)	LOD (mg kg^−1^)	LOQ (mg kg^−1^)	Recovery (%)
Beverage	Nitrate	*y* = 0.1008*x* + 0.0353	0.9993	2.5–15	0.5	1.4	95–101
	Nitrite	*y* = 0.1665*x* + 0.0981	0.9967	2.5–15	0.5	1.4	98–109

Fruit	Nitrate	*y* = 0.1032*x* + 0.0541	0.9979	2.5–15	1	2.8	91–99
	Nitrite	*y* = 0.1214*x* − 0.0508	0.9956	2.5–15	1	2.8	97–104

Vegetable	Nitrate	*y* = 0.1195*x* + 0.0243	0.9961	2.5–15	1	2.8	90–96
	Nitrite	*y* = 0.1415*x* − 0.0181	0.9949	2.5–15	1	2.8	98–102

Stew	Nitrate	*y* = 0.1168*x* + 0.1735	0.9917	2.5–15	0.5	1.4	89-90
	Nitrite	*y* = 0.1262*x* − 0.0597	0.9883	2.5–15	0.5	1.4	92–96
